# Perceived stress among suspected patients during the COVID-19 outbreak in Tunisia

**DOI:** 10.1192/j.eurpsy.2021.1743

**Published:** 2021-08-13

**Authors:** A. Zouari, J. Ben Thabet, A. Guermazi, J. Aloulou, R. Hammami, H. Ben Ayed, A. Sallemi, C. Marrekchi, S. Hdiji, I. Gargouri, M. Kassis, M. Turki, S. Kammoun, M.L. Masmoudi

**Affiliations:** 1 Department Of Paychiatry C, Hedi chaker hospital, Sfax, Tunisia; 2 Psychiatry C Department, Hedi chaker University hospital, sfax, Tunisia; 3 Psychiatry (b), Hedi Chaker University hospital, sfax, Tunisia; 4 Cardiology Department, Hedi chaker University hospital, sfax, Tunisia; 5 Community Health And Epidemiology Department, HediChaker Hospital, sfax, Tunisia; 6 Histology Department, Hedi chaker University hospital, sfax, Tunisia; 7 Infectiology Department, Hedi chaker University hospital, sfax, Tunisia; 8 Hematology Department, Hedi chaker University hospital, sfax, Tunisia; 9 Occuaptional Department, HediChaker Hospital, sfax, Tunisia; 10 Pharmacy, HediChaker Hospital, tunis, Tunisia; 11 Pneumology, HediChaker Hospital, sfax, Tunisia

**Keywords:** perceived stress, suspected patients, COVID-19

## Abstract

**Introduction:**

Widespread outbreaks of infectious disease, such as COVID-19, are associated with psychological distress and symptoms of mental illness especially for patients with suggestive symptoms.

**Objectives:**

Predict the prevalence of perceived stress and study associated factors among patients with suspected COVID-19 infection.

**Methods:**

A cross sectional study was conducted between April and May 2020. Patients consulting the sorting box at the Hedi Chaker Hospital of Sfax and declared suspect to be infected by COVID-19 were invited to participate in our study after given their cosent. Perceived Stress Scale-10 was used to evaluate prevalence of perceived stress.

**Results:**

In total, 149 participants participated. The mean age was 38.8±15.39 years. Medical or surgical history and psychiatric history were identified respectively in 30,2% and 12.1% of participants. Among all respondents, 74.5% took a nasopharyngeal swab to look for COVID-19 and only 6.4% had a positive test. Close contact with someone with a positive COVID-19 infection was found in 8.05%. Several participants (79,2%) expressed fear of transmitting the disease to their family members. The mean of the PSS-10 score was 11.97±9.83. Moderate to severe perceived stress was found in 44.3% of patients. Significantly higher scores were observed among participants with a positive pharyngeal swab for COVID-19 as well as those who perceived worry of transmetting the disease. No significant differences in perceived stress’ scores according to socio-demographic data.

**Conclusions:**

Perceived stress was high among patients with suspected COVID-19 infection. Perceiving worry of transmetting the disease and having a positive pharyngeal swab for COVID-19 were the principal risk factors.

**Disclosure:**

No significant relationships.

